# Effects of Vitamin E on Growth Performance, Biochemical Indices, Antioxidant Capacity, and Ovarian Development in Female Chinese Sturgeon (*Acipenser sinensis*) Broodstock

**DOI:** 10.3390/ani16132051

**Published:** 2026-07-03

**Authors:** Pei Chen, Jing Yang, Baifu Guo, Yacheng Hu, Hongtao Huang, Wei Jiang, Zhiyuan Li, Kan Xiao

**Affiliations:** Hubei Key Laboratory of Three Gorges Projects for Fishes Conservation, Chinese Sturgeon Research Institute of China Three Gorges Corporation, Yichang 443100, China; chenpei879368301@126.com (P.C.); yang_jing7@ctg.com.cn (J.Y.); guo_baifu@ctg.com.cn (B.G.); hu_yacheng@ctg.com.cn (Y.H.); huang_hongtao@ctg.com.cn (H.H.); jiang_wei6@ctg.com.cn (W.J.)

**Keywords:** anadromous species, α-tocopherol acetate, growth, antioxidant status, sexual maturation

## Abstract

This study investigated the effects of dietary vitamin E (DL-α-tocopherol acetate) supplementation on growth performance, biochemical indices, antioxidant capacity, and ovarian development in female Chinese sturgeon (*Acipenser sinensis*) broodstock. A total of 180 healthy second-generation individuals (initial weight: 72.63 ± 3.64 kg) were randomly distributed into three groups and fed isonitrogenous and isolipidic diets supplemented with 0 (V0), 1 (V1), or 2 (V2) g/kg vitamin E for 14 months. At the end of the trial, fish in the V2 group exhibited significantly higher final body weight, body length, and girth, demonstrating superior growth performance compared to the V0 and V1 groups (*p* < 0.05). Plasma antioxidant capacity was significantly enhanced in the V2 group, as indicated by elevated activities of catalase, superoxide dismutase, and total antioxidant capacity, alongside reduced levels of reactive oxygen species and malondialdehyde (*p* < 0.05). Biochemical analysis revealed that vitamin E supplementation significantly decreased plasma glucose, urea nitrogen, and alkaline phosphatase levels (*p* < 0.05), with the V2 group showing a notable increase in high-density lipoprotein cholesterol (*p* < 0.05). Ultrasonographic examination demonstrated that ovarian thickness in both transverse and longitudinal sections was significantly increased in the V2 group (*p* < 0.05). This morphological improvement was corroborated by significantly higher plasma levels of estradiol and vitellogenin in the supplemented groups, particularly in the V2 group. In conclusion, the current study revealed that dietary 2 g/kg vitamin E supplementation not only promoted growth performance and enhanced antioxidant capacity but also effectively stimulated ovarian development by modulating key reproductive hormones in female Chinese sturgeon broodstock.

## Simple Summary

The Chinese sturgeon, a critically endangered ancient species native to the Yangtze River, faces severe conservation challenges due to the failure of natural reproduction in the wild. Captive breeding programs are essential for its survival, but female broodstock often experience arrested ovarian development, which limits artificial propagation success. In this study, we investigated the effects of dietary vitamin E on growth performance, biochemical indices, antioxidant capacity, and ovarian development in Chinese sturgeon broodstock. Results showed that dietary 2 g/kg vitamin E supplementation significantly improved growth performance, enhanced antioxidant capacity, and promoted ovarian development in Chinese sturgeon broodstock.

## 1. Introduction

The Chinese sturgeon (*Acipenser sinensis*), an ancient anadromous species, serves as a critical bioindicator for the ecological integrity of the Yangtze River system [1]. However, the interruption of natural reproduction in consecutive years, driven by multiple environmental stressors, has posed severe threats to its survival [2]. Consequently, artificial propagation and stocking have become pivotal strategies for population restoration [3]. Nevertheless, a major bottleneck hindering these conservation efforts is the low sexual maturation rate in captive female broodstock. Ovarian development in this species is frequently arrested at stage II, preventing progression to the vitellogenic stages (III and IV) [4,5].

Nutritional intervention has emerged as a promising approach to overcoming this developmental stagnation. While previous studies have demonstrated that high-lipid diets and specific nutrients, such as arachidonic acid (ARA), can facilitate ovarian maturation through steroid hormone regulation [5,6,7,8], research on functional feed additives in Chinese sturgeon reproductive nutrition remains scarce. Vitamin E (α-tocopherol), a potent lipid-soluble antioxidant essential for preventing the polyunsaturated fatty acid peroxidation in biological membranes [9,10], has been extensively documented to enhance growth performance, antioxidant capacity, and reproductive function in various teleost species, such as grass carp (*Ctenopharyngodon idellus*) [11,12], tilapia (*Oreochromis niloticus*) [13,14], yellow catfish (*Pelteobagrus fulvidraco*) [15], largemouth bass (*Micropterus salmoides*) [16,17], and Arabian yellowfin seabream (*Acanthopagrus arabicus*) [18]. Beyond its classical antioxidant role, vitamin E promotes sex hormone secretion, increases the gonadosomatic index, and improves spawning performance and egg quality [17,18,19].

Although vitamin E has been extensively studied in teleosts, research specifically focusing on sturgeons remains relatively limited. Previous investigations have primarily been conducted on species such as the Adriatic sturgeon (*Acipenser naccarii*) [20], lake sturgeon (*Acipenser fulvescens* R.) [21], beluga sturgeon (*Huso huso* L.) [22], and stellate sturgeon (*Acipenser stellatus*). More recently, studies on juvenile Chinese sturgeon have demonstrated that dietary supplementation with 1 g/kg vitamin E enhances growth performance and antioxidant capacity while upregulating gene expression related to the hypothalamic–pituitary–gonadal (HPG) axis [23,24]. Despite these advances, the effects of vitamin E on female Chinese sturgeon broodstock remains poorly understood. Thus, this study aims to evaluate the effects of graded levels of vitamin E on growth performance, antioxidant status, plasma biochemical indices, and ovarian development in female Chinese sturgeon broodstock. The findings are expected to provide a scientific basis for optimizing dietary formulations to increase sexual maturation rate and support conservation efforts for this critically endangered species.

## 2. Material and Methods

Throughout the experimental duration, the fish were handled in accordance with the Laboratory Animal Welfare Guidelines of the Hubei Key Laboratory of Three Gorges Projects for Fish Conservation, Chinese Sturgeon Research Institute of China Three Gorges Corporation.

### 2.1. Experimental Diets

Three experimental diets were prepared with graded levels of vitamin E (0, 1, and 2 g/kg) [23,24,25] designated as V0 (control), V1, and V2 groups, respectively. The diets were processed into 10–12 mm sinking extruded pellets by Tianmen Haida Feed Co., Ltd. (Jingzhou, China). DL-α-tocopheryl acetate (50% purity) was purchased from Jilin Baisha Pharmaceutical Co., Ltd. (Jilin, China), while Tianmen Haida Feed Co., Ltd., provided other ingredients. The actual vitamin E concentrations in the diet were detected by high-performance liquid chromatography to be 14 (control group), 1023, and 2018 mg/kg, respectively. All diets underwent air drying at ambient temperature and were subsequently stored at −20 °C until required. The formulation and nutrient composition of the diets are presented in Table 1.

### 2.2. Feeding Trial

Second-generation (F2) female Chinese sturgeon bred in 2011 at the Chinese Sturgeon Research Institute (Yichang, China) were used as experimental subjects. Prior to the trial, fish were acclimated for one week using the basal diet (V0) at a daily feeding rate of 0.2% body weight (BW/d).

A total of 180 fish (approximately 13 years old, initial total length of 188.84 ± 2.99 cm, girth of 90.24 ± 1.92 cm, and body weight of 72.63 ± 3.64 kg) with gonads at developmental stage II were randomly allocated into nine independent concrete tanks (12 m diameter × 2.2 m water depth), arranged into three groups with three replicates per group. The fish were fed daily at 09:00 for a 14-month trial period (November 2023 to December 2024). Feeding rates were adjusted between 0.1% and 0.4% BW/d based on fish growth and water temperature. During the experiment, water temperature was maintained between 12 °C and 29 °C, with a cooling system activated when the temperature exceeded 30 °C. Dissolved oxygen was maintained >7 mg/L, and ammonia nitrogen < 0.2 mg/L. The fish were maintained under a natural light–dark cycle. The experiment utilized a flow-through system; the tanks were drained once daily, achieving a daily water exchange rate of 2–3 tank volumes.

### 2.3. Sample Collection and Calculation of Growth Indices

At the conclusion of the trial, fish were starved for 24 h. The body weight, total length, and girth of fish were measured individually. Five fish from each tank were randomly selected for blood sampling. Approximately 3 mL of blood was rapidly collected from the caudal vein using vacuum tubes containing sodium heparin. The samples were centrifuged at 3000× *g* for 10 min at 4 °C to obtain plasma for the analysis of biochemical parameters. Body length was defined as the distance from the snout tip to the end of the caudal vertebrae, and girth was defined as the maximum circumference of the body. The growth indices were calculated as follows:

Body length growth rate (BGR, %) = 100 × (Final body length − Initial body length)/Initial body length

Girth growth rate (GGR, %) = 100 × (Final girth − Initial girth)/Initial girth

Body weight gain rate (WGR, %) = 100 × (Final body weight − Initial body weight)/Initial body weight

Survival rate (SR, %) = 100 × Number of surviving fish/Number of fish at the start of the trial

Feed conversion ratio (FCR) = Feed intake (kg)/weight gain (kg)

Protein efficiency ratio (PER) = Weight gain (kg)/protein intake (kg)

### 2.4. Ovarian Development Status

**Figure 1:**
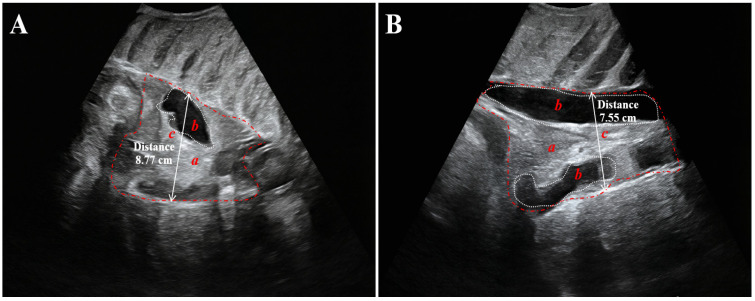
Ultrasound images of the ovary in Chinese sturgeon, *Acipenser sinensis*. (**A**) Transverse view of the ovary; (**B**) Longitudinal view of the ovary. a: Ovarian region (area marked by red dashed line); b: Ovarian fat region (area marked by white dashed line); c: Ovarian thickness (distance between white arrows, cm).

A Terason 3000™ (5–2 MHz) portable ultrasound scanner (Burlington, MA, USA) was employed to perform ultrasonography on the ventral surface of the Chinese sturgeon, focusing on the region between the third and fourth scutes (counted from the caudal to the cranial direction). Gonad development was assessed using ultrasound imaging, in which the shape and echogenicity of the gonad and gonadal fat, the thickness of the whole reproductive organs (*d*) and the proportion of the ovary to the whole reproductive organs (*po*) were the main ultrasonographic characteristics, as those are the best indicators of sex and gonadal maturity. For more detailed methods, please refer to Du et al. [26] and Guo et al. [27]. Ultrasound images were acquired to measure ovarian thickness and to determine the stage of ovarian development. Ovarian thickness was quantified from both transverse and longitudinal views using the ImageJ software system (version 1.8.0, NIH, Bethesda, MD, USA) (Figure 1).

### 2.5. Nutrient Composition Analysis

The chemical compositions of the feed were analyzed in accordance with the standard methodologies established by the Association of Official Analytical Chemists [28]. Moisture content was assessed by drying samples at 105 °C until reaching a constant weight. Crude protein content was determined via the Kjeldahl method, crude lipid through Soxhlet extraction using diethyl ether, and ash content by incineration in a muffle furnace at 550 °C for 4 h.

### 2.6. Plasma Biochemical Indices and Sex Hormones

Assay kits for the determination of total protein (TP), glucose, triglyceride (TG), total cholesterol (TC), high-density lipoprotein cholesterol (HDL-C), low-density lipoprotein cholesterol (LDL-C), aspartate aminotransferase (AST), alanine aminotransferase (ALT), alkaline phosphatase (AKP), acid phosphatase (ACP), urea nitrogen (BUN), total bile acid (TBA), vitamin E, total antioxidant capacity (T-AOC), reduced glutathione (GSH), catalase (CAT), superoxide dismutase (SOD), malondialdehyde (MDA) were all purchased from Nanjing Jiancheng Co., Ltd. (Jiangshu, China). Assay kits for the detection of reactive oxygen species (ROS), gonadotropin-releasing hormone (GnRH), follicle-stimulating hormone (FSH), luteinizing hormone (LH), estradiol (E_2_), and vitellogenin (VTG) were purchased from Keerda Biotechnology Co., Ltd. (Wuhan, China). All assays were performed following the manufacturer’s instructions. All physiological parameters were assessed with a microplate reader (PowerWave XS2, BioTek, Minneapolis, MN, USA) according to the guidelines.

### 2.7. Statistical Analysis

Growth data were analyzed with the independent tank as the experimental unit for statistical analyses. Blood parameters and ovarian thickness were analyzed with the individual fish as the experimental unit for statistical analyses. Statistical analyses were performed with SPSS software version 26.0 (IBM Corp., Armonk, NY, USA). Before the statistical analysis, the data were checked for homogeneity of variances and normality of distribution. Data normality was assessed using the Shapiro–Wilk test, and homogeneity of variance was tested using Levene’s test. A one-way analysis of variance (ANOVA) followed by Duncan’s post hoc test was conducted to identify significant differences (*p* < 0.05) among treatment groups. Results are presented as mean ± standard error.

## 3. Results

### 3.1. Growth Performance

The effects of dietary vitamin E on the growth performance of Chinese sturgeon are summarized in Table 2. After the 14-month feeding trial, fish fed the V2 diet exhibited significantly superior growth compared to the V0 and V1 groups (*p* < 0.05). Specifically, the final body length, final girth, and final body weight were markedly higher in the V2 group (*p* < 0.05). Consequently, the BGR, GGR, and WGR were significantly enhanced by the 0.2% vitamin E supplementation (*p* < 0.05). However, dietary vitamin E had no significant effect on FCR and PER (*p* > 0.05). The SR remained at 100% across all experimental groups (*p* > 0.05).

### 3.2. Plasma Biochemical Parameters

The plasma biochemical indices are detailed in Table 3. Compared to the control, the V1 and V2 groups displayed significantly lower levels of glucose, BUN, and AKP (*p* < 0.05). The plasma HDL-C levels were significantly higher in the V2 group compared to the V0 and V1 groups (*p* < 0.05). Conversely, other metabolic parameters (TP, TG, TC, LDL-C) and liver function markers (TBA, ACP, AST, ALT) were not significantly affected by the dietary treatments (*p* > 0.05).

### 3.3. Plasma Antioxidant Capacity and Vitamin E Contents

As presented in Table 4, dietary vitamin E supplementation improved the plasma antioxidant status and vitamin E contents of Chinese sturgeon. Compared to the V0 group, both the V1 and V2 groups exhibited significantly lower levels of ROS and MDA (*p* < 0.05), indicating a reduction in oxidative stress. In particular, plasma T-AOC and SOD activities were significantly higher in the V2 group compared to both the V0 and V1 groups (*p* < 0.05). Moreover, CAT activity was significantly elevated in both supplemented groups (V1 and V2) compared to the control (*p* < 0.05), with the highest activity observed in the V2 group. Plasma GSH levels remained unaffected by the dietary treatments (*p* > 0.05). Consistent with the antioxidant enzyme responses, plasma vitamin E contents were dose-dependently increased, with the V2 group showing the highest levels (*p* < 0.05).

### 3.4. Ovarian Development and Reproductive Hormones

The influence of vitamin E on ovarian development and plasma sex hormones is shown in Table 5 and Table 6. Ultrasonography revealed that the V2 group exhibited significantly greater ovarian thickness in both transverse and longitudinal sections compared to the V0 group (*p* < 0.05), while the V1 group showed an intermediate improvement (Table 5). Importantly, plasma sex hormone analysis (Table 6) corroborated the morphological findings. The V2 group showed significantly higher plasma E_2_ levels compared to the V0 and V1 groups (*p* < 0.05). Furthermore, VTG concentrations were significantly upregulated in both supplemented groups, with the V2 group exhibiting the highest levels. However, no significant differences were observed in the plasma concentrations of GnRH, FSH, or LH among the three groups (*p* > 0.05).

## 4. Discussion

### 4.1. Effects of Vitamin E on Growth Performance

The present study demonstrated that dietary 2 g/kg vitamin E supplementation significantly enhanced the growth performance of female Chinese sturgeon broodstock. These findings aligned with previous research on Nile tilapia [19] and pindani (*Pseudotropheus socolofi*) [29]. However, contrasting results have been reported in other species. For instance, Tao et al. [30] found that dietary vitamin E supplementation at 200 mg/kg did not significantly affect growth performance in female Nile tilapia broodstock, while Najafabadi et al. [18] observed no notable effects on growth parameters in Arabian yellowfin seabream broodstock fed 250 mg/kg vitamin E.

The present study revealed a higher dietary vitamin E requirement for adult Chinese sturgeon broodstock (2 g/kg) compared to the 1 g/kg requirement previously established for one-year-old juvenile conspecifics [23,24]. This differential requirement can be primarily explained by two key physiological factors: metabolic scaling effects and reproductive energy allocation. First, metabolic scaling theory posits that larger organisms require disproportionately higher concentrations of fat-soluble nutrients relative to their body mass [31]. As a large-bodied, long-lived chondrostean fish, the adult Chinese sturgeon possesses extensive lipid storage depots in both muscular and visceral tissues [5]. The volume of these lipid-rich tissues increases allometrically with body size, necessitating higher vitamin E concentrations to achieve adequate tissue saturation and maintain antioxidant protection [9,10]. Consequently, the dietary concentration required to achieve effective plasma and tissue saturation in broodstock (average body weight: 72.63 kg) substantially exceeds that needed for juveniles. Second, broodstock fish face the dual metabolic challenge of maintaining somatic tissues while simultaneously supporting the energetically demanding process of vitellogenesis [32]. This increased metabolic activity elevates basal metabolic rate and consequently enhances ROS production [33]. The elevated vitamin E levels in the V2 group likely mitigated this oxidative stress by enhancing antioxidant defense mechanisms, thereby protecting cellular integrity and enabling sustained growth despite the physiological demands of reproduction.

### 4.2. Effects of Vitamin E on Plasma Antioxidant Capacity and Biochemical Indices

Vitamin E functions as a primary lipid-soluble antioxidant by scavenging free radicals through its ability to donate hydrogen atoms to peroxyl radicals, thereby terminating chain reactions in biological membranes [9]. Within the antioxidant defense system, enzymatic components (such as SOD and CAT) work synergistically with non-enzymatic antioxidants (including vitamin E) to neutralize ROS and maintain cellular redox homeostasis [33]. MDA, a stable end-product of lipid peroxidation, serves as a reliable biomarker for oxidative damage to cellular membranes [34]. In the present study, dietary supplementation with 2 g/kg vitamin E (the V2 group) significantly reduced plasma ROS and MDA concentrations while simultaneously enhancing the level of T-AOC and the activities of SOD and CAT in Chinese sturgeon broodstock. These findings corroborated that vitamin E effectively mitigated oxidative stress in this species. Similar protective effects have been documented in other fish species: female Nile tilapia broodstock exhibited reduced plasma MDA and elevated SOD activity when fed 70–200 mg/kg vitamin E [30], while Arabian yellowfin seabream broodstock showed enhanced T-AOC, CAT, and SOD activities with concurrent reduction in serum lipid peroxidation following supplementation with 100 mg/kg vitamin E [18]. Comparable responses have also been reported in juvenile teleosts, including grass carp [11], yellow catfish [15], tilapia [19], and Chinese sturgeon [23]. The absence of significant differences in GSH levels across treatment groups suggested that the glutathione-dependent pathway was not the primary mechanism by which vitamin E exerted its antioxidant effects in Chinese sturgeon broodstock. Alternatively, GSH synthesis capacity likely remained adequate under experimental conditions without becoming rate-limiting [35]. It is worth highlighting that plasma vitamin E concentrations were significantly elevated in both V1 and V2 groups, confirming efficient dietary absorption and systemic retention.

Beyond its canonical antioxidant role, vitamin E modulated systemic metabolism, as evidenced by alterations in key plasma biochemical indices. As a recognized biomarker of stress, plasma glucose typically rises to meet increased energy demands [36]. The present results showed that vitamin E significantly reduced glucose levels in female Chinese sturgeon compared to the control. This is corroborated by a study on Nile tilapia, which demonstrated that dietary vitamin E (0.1 g/kg) effectively lowered glucose concentrations [37]. These findings implied that dietary vitamin E provided supplementary stress-reducing benefits for Chinese sturgeon. Additionally, the decreased plasma BUN levels in the V1 and V2 groups pointed to enhanced nitrogen utilization efficiency mediated by vitamin E [38]. The decline in AKP activity, an enzyme marker associated with hepatic metabolism, potentially indicated a reduced metabolic burden on the liver or enhanced hepatic function [39]. Notably, the significant elevation in plasma HDL-C observed in the V2 group suggested enhanced reverse cholesterol transport capacity [40]. This metabolic adaptation not only supports cardiovascular health but also provides essential cholesterol substrates for ovarian steroidogenesis—a phenomenon previously documented in tilapia [14] and largemouth bass [17]. Therefore, vitamin E functioned as both a direct antioxidant and systemic metabolic regulator in female Chinese sturgeon broodstock, creating favorable physiological conditions that supported reproductive health.

### 4.3. Effects of Vitamin E on Ovarian Development

Vitamin E plays a critical role in the reproductive physiology of aquatic animals [10]. Adequate dietary vitamin E supplementation have been shown to promote steroid hormone secretion and yolk deposition [41]. The present study demonstrated that dietary 2 g/kg vitamin E supplementation significantly enhanced ovarian development in Chinese sturgeon broodstock. This enhancement was evidenced by increased ovarian thickness in both transverse and longitudinal dimensions, coupled with elevated plasma E_2_ and VTG levels. The observed increase in ovarian thickness correlated strongly with elevated plasma E_2_ concentrations in the V2 group, suggesting that vitamin E enhanced steroidogenesis directly within the ovarian follicles. This finding alignd with research in Nile tilapia, where 400 mg/kg vitamin E supplementation significantly increased plasma E_2_ levels and promoted vitellogenesis by upregulating hepatic VTG synthesis [42]. Similarly, Tao et al. [30] reported that 200 mg/kg vitamin E effectively mitigated impaired ovarian development in Nile tilapia fed olive oil-based diets by restoring E_2_ production and enhancing the antioxidant capacity of ovarian tissues. The significant elevation in plasma VTG levels, particularly in the V2 group, further supported a hormone-mediated mechanism of action. As a female-specific yolk precursor protein regulated by E_2_, VTG serves as a sensitive biomarker for ovarian development and vitellogenic activity [10,43]. Vitamin E’s ability to enhance VTG synthesis likely stems from its dual role in protecting ovarian cells from oxidative damage and facilitating cholesterol transport for steroid hormone production [41]. This mechanism is consistent with findings in Arabian yellowfin seabream, where 250 mg/kg dietary vitamin E significantly increased ovarian VTG content and improved egg quality [18].

Notably, the lack of significant changes in pituitary gonadotropins (GnRH, FSH, and LH) in the present study suggested that the regulatory effects of vitamin E did not primarily stem from the hypothalamic–pituitary axis. Instead, the pronounced upregulation of downstream ovarian hormones (E_2_) and VTG implied that vitamin E exerted its protective role in the ovary and liver rather than through a neuroendocrine stimulatory function [41]. Specifically, vitamin E appears to facilitate steroidogenesis directly within the gonads [44] and VTG synthesis in the liver [45]. This is supported by our finding that vitamin E supplementation enhanced the bioavailability of cholesterol—the precursor for steroidogenesis—as evidenced by increased HDL-C levels. This aligned with observations in Japanese flounder (*Paralichthys olivaceus*) [46], where vitamin E accumulation in gonads was functionally linked to vitellogenesis. While some in vitro studies had suggested that vitamin E could stimulate gonadotropin expression in primary pituitary cells of turbot (*Scophthalmus maximus*) [47], our in vivo results highlighted that under physiological conditions, its primary mode of action might be the direct modulation of peripheral steroidogenic and vitellogenic pathways.

While this study provided robust evidence for the beneficial effects of vitamin E on ovarian development through ultrasonographic and hormonal assessments, the absence of histological validation to confirm specific follicular maturation stages represented a study limitation. Future investigations incorporating histopathological analysis would further elucidate the structural changes underlying the observed improvements in ovarian morphology and reproductive hormone profiles.

## 5. Conclusions

The current study revealed that dietary 2 g/kg vitamin E supplementation significantly improved the growth performance and enhanced antioxidant capacity of female Chinese sturgeon broodstock. Dietary 2 g/kg vitamin E supplementation significantly promoted ovarian development, as evidenced by increased ovarian thickness and elevated plasma E_2_ and VTG levels. These findings provide a theoretical basis for the nutritional regulation of gonadal development in female Chinese sturgeon. Future studies should employ more graded levels to pinpoint the exact requirement threshold, while also elucidating the molecular mechanisms underlying the effects of vitamin E on the HPG axis.

## Figures and Tables

**Table 1:** Formulation and nutrient compositions of experimental diets.

Ingredients (g/kg)	V0	V1	V2
Fish meal	400	400	400
Chicken powder meal	150	150	150
Cottonseed protein concentrate	150	150	150
Wheat flour	180	180	180
Fish oil	40	40	40
Soybean oil	30	30	30
Lecithin oil	15	15	15
Vitamin and mineral premix ^a^	12	12	12
Ca(H_2_PO_4_)_2_	8	8	8
Choline chloride (60%)	4	4	4
Bentonite	11	9	7
Vitamin E (50%)		2	4
Total	1000	1000	1000
Analyzed chemical composition (dry matter basis, %)			
Moisture	6.45	6.67	6.56
Crude ash	11.65	11.42	11.46
Crude protein	50.21	49.97	50.11
Crude lipid	13.35	13.63	13.45

^a^ Vitamin premix (mg/kg diet): Vitamin A 20; Vitamin D_3_ 10; Vitamin K_3_ 20; Vitamin B_1_ 10; Vitamin B_2_ 15; Vitamin B_6_ 15; Vitamin B_12_ (1%) 8; Ascorbic acid (35%) 1000; Calcium pantothenate 40; Niacinaminde 100; Inositol 200; Biotin (2%) 2; Folic acid 10; Corn gluten meal 550. Mineral premix (mg/kg diet): CuSO_4_·5H_2_O 10; FeSO_4_·H_2_O 300; ZnSO_4_·H_2_O 200; MnSO_4_·H_2_O 100; KI (10%) 80; CoCl_2_·6H_2_O (10%Co) 5; Na_2_SeO_3_ (10% Se) 10; MgSO_4_·5H_2_O 2000; NaCl 2100; Zeolite 4995; Antioxidant 200.

**Table 2:** Effects of vitamin E on the growth performance of Chinese sturgeon, *Acipenser sinensis*.

	V0	V1	V2
Final body length (cm)	198.95 ± 2.71 ^a^	196.75 ± 2.11^a^	206.06 ± 2.26 ^b^
Final girth (cm)	93.89 ± 1.72 ^a^	94.22 ± 1.25 ^a^	98.50 ± 1.30 ^b^
Final body weight (kg)	83.11 ± 3.47 ^a^	82.33 ± 2.74 ^a^	93.35 ± 2.83 ^b^
BGR (%)	5.35 ± 1.43 ^a^	4.19 ± 1.12 ^a^	9.12 ± 1.20 ^b^
GGR (%)	4.05 ± 1.91 ^a^	4.41 ± 1.39 ^a^	9.15 ± 1.44 ^b^
WGR (%)	14.29 ± 4.77 ^a^	13.32 ± 3.77 ^a^	28.37 ± 3.89 ^b^
FCR	3.42 ± 0.48	3.53 ± 0.41	3.03 ± 0.38
PER	0.70 ± 0.09	0.72 ± 0.09	0.86 ± 0.11
SR (%)	100	100	100

BGR, Body length growth rate; GGR, Girth growth rate; WGR, Body weight gain rate; FCR, Feed conversion ratio; PER, Protein efficiency ratio; SR, Survival rate. Within the same row, values with different superscripts are significantly different (*p* < 0.05).

**Table 3:** Effects of vitamin E on the plasma biochemical parameters of Chinese sturgeon, *Acipenser sinensis*.

	V0	V1	V2
TP g/L	28.92 ± 2.34	29.77 ± 2.57	30.93 ± 2.21
Glucose mmol/L	8.55 ± 0.82 ^b^	6.52 ± 0.55 ^a^	6.08 ± 0.56 ^a^
TG mmol/L	1.45 ± 0.31	1.45 ± 0.21	1.25 ± 0.19
TC mmol/L	1.52 ± 0.18	1.51 ± 0.12	1.64 ± 0.14
HDL-C mmol/L	0.33 ± 0.04 ^a^	0.36 ± 0.05 ^a^	0.57 ± 0.06 ^b^
LDL-C mmol/L	0.67 ± 0.05	0.81 ± 0.07	0.66 ± 0.05
BUN μmol/L	250.66 ± 21.42 ^b^	168.25 ± 16.53 ^a^	167.58 ± 16.37 ^a^
TBA μmol/L	68.23 ± 16.99	59.36 ± 12.87	54.58 ± 8.51
AKP mg/L	47.49 ± 1.90 ^b^	34.47 ± 3.04 ^a^	31.78 ± 1.42 ^a^
ACP mg/L	136.60 ± 17.50	147.58 ± 17.33	157.64 ± 45.94
AST U/L	7.16 ± 1.14	8.94 ± 1.08	8.78 ± 0.76
ALT U/L	3.66 ± 0.68	4.08 ± 0.74	2.60 ± 0.26

TP, total protein glucose; TG, triglyceride; TC, total cholesterol; HDL-C, high-density lipoprotein cholesterol; LDL-C, low-density lipoprotein cholesterol; BUN, urea nitrogen; TBA, total bile acid; AKP, alkaline phosphatase; ACP, acid phosphatase; AST, aspartate aminotransferase; ALT, alanine aminotransferase. Within the same row, values with different superscripts are significantly different (*p* < 0.05).

**Table 4:** Effects of vitamin E on plasma antioxidant parameters and vitamin E contents of Chinese sturgeon, *Acipenser sinensis*.

	V0	V1	V2
ROS μg/L	58.62 ± 3.40 ^b^	47.54 ± 3.20 ^a^	43.92 ± 3.34 ^a^
T-AOC μmol/L	268.76 ± 12.67 ^a^	289.15 ± 8.09 ^a^	321.92 ± 5.75 ^b^
GSH μmol/L	188.86 ± 7.92	186.41 ± 13.28	179.98 ± 4.88
CAT U/mL	1.92 ± 0.24 ^a^	2.72 ± 0.22 ^b^	2.68 ± 0.23 ^b^
SOD U/mL	24.94 ± 3.07 ^a^	25.08 ± 2.41 ^a^	36.22 ± 4.48 ^b^
MDA μmol/L	31.03 ± 1.57 ^b^	21.12 ± 1.77 ^a^	22.83 ± 1.52 ^a^
Vitamin E mg/L	3.65 ± 0.47 ^a^	11.67 ± 0.82 ^b^	22.35 ± 1.63 ^c^

ROS, reactive oxygen species; T-AOC, total antioxidant capacity; GSH, reduced glutathione; CAT, catalase; SOD, superoxide dismutase; MDA, malondialdehyde. Within the same row, values with different superscripts are significantly different (*p* < 0.05).

**Table 5:** Effects of vitamin E on the ovarian thickness of Chinese sturgeon, *Acipenser sinensis*.

	V0	V1	V2
Transverse section (cm)	7.99 ± 0.50 ^a^	9.72 ± 0.48 ^b^	10.22 ± 0.44 ^b^
Longitudinal section (cm)	7.56 ± 0.48 ^a^	8.37 ± 0.37 ^ab^	9.00 ± 0.40 ^b^

Within the same row, values with different superscripts are significantly different (*p* < 0.05).

**Table 6:** Effects of vitamin E on the plasma sex hormones of Chinese sturgeon, *Acipenser sinensis*.

	V0	V1	V2
GnRH ng/L	54.85 ± 4.75	44.99 ± 7.48	47.19 ± 4.25
FSH U/L	3.00 ± 0.38	3.26 ± 0.77	2.89 ± 0.43
LH ng/L	22.89 ± 0.54	23.23 ± 0.45	22.06 ± 0.46
E_2_ ng/L	55.71 ± 0.69 ^a^	58.38 ± 0.87 ^a^	63.57 ± 1.30 ^b^
VTG mg/L	210.04 ± 14.18 ^a^	260.40 ± 22.70 ^ab^	278.74 ± 16.75 ^b^

GnRH, gonadotropin-releasing hormone; FSH, follicle-stimulating hormone; LH, luteinizing hormone; E_2_, estradio; VTG, vitellogenin. Within the same row, values with different superscripts are significantly different (*p* < 0.05).

## Data Availability

The data that support the findings of this study are available from the corresponding author upon reasonable request.
